# Continuous Force Decoding from Local Field Potentials of the Primary Motor Cortex in Freely Moving Rats

**DOI:** 10.1038/srep35238

**Published:** 2016-10-21

**Authors:** Abed Khorasani, Nargess Heydari Beni, Vahid Shalchyan, Mohammad Reza Daliri

**Affiliations:** 1 Neuroscience and Neuroengineering Research Lab., Department of Biomedical Engineering, School of Electrical Engineering, Iran University of Science and Technology (IUST), Narmak, 16846-13114 Tehran, Iran

## Abstract

Local field potential (LFP) signals recorded by intracortical microelectrodes implanted in primary motor cortex can be used as a high informative input for decoding of motor functions. Recent studies show that different kinematic parameters such as position and velocity can be inferred from multiple LFP signals as precisely as spiking activities, however, continuous decoding of the force magnitude from the LFP signals in freely moving animals has remained an open problem. Here, we trained three rats to press a force sensor for getting a drop of water as a reward. A 16-channel micro-wire array was implanted in the primary motor cortex of each trained rat, and obtained LFP signals were used for decoding of the continuous values recorded by the force sensor. Average coefficient of correlation and the coefficient of determination between decoded and actual force signals were r = 0.66 and R^2^ = 0.42, respectively. We found that LFP signal on gamma frequency bands (30–120 Hz) had the most contribution in the trained decoding model. This study suggests the feasibility of using low number of LFP channels for the continuous force decoding in freely moving animals resembling BMI systems in real life applications.

Advent of invasive microelectrodes in brain machine interface (BMI) systems has provided a practical tool for restoration of movements in individuals with paralyzed limbs^1^,^2^. In these motor BMIs, movement is decoded from cortical neurons and then a proper motor command is produced to move a real body part (exoskeleton, muscle innervation) or an artificial effector similar to healthy subjects controlling their body parts. The spiking activity recorded with high density multi-electrode array from primary motor cortex (M1) can provide the highest-resolution brain signals in both space and time domains. However, local field potential (LFP) signals representing mainly synaptic activity of local neurons would be a better choice than spikes in practical BMI systems, because of both stability in long periods of time and simplicity of the extraction^3^. On the other hand, unlike semi invasive methods like electrocorticography (ECoG) recordings, LFP has a higher signal to noise ratio and spatial resolution. Therefore, LFP would be a more informative choice for decoding of movement parameters in BMIs.

Recent studies have shown that different movement-related parameters such as direction, position and velocity can be inferred from multichannel LFPs as precisely as spiking activities^4^,^5^,^6^,^7^,^8^. For instance, reaching direction can be decoded from the spectral features of multichannel LFPs^9^,^10^. Furthermore, continuous movement parameters such as position and velocity in 3-D or 2-D space can be decoded from multichannel LFPs recorded from motor cortex^4^,^8^,^11^.

All of the aforementioned studies have focused on the decoding of kinematic parameters. However, in common everyday tasks like grasping an object or pressing a button, accurate decoding of force amplitude is also critical. Nevertheless, a few studies have focused on the continuous decoding of force from cortical signals^12^,^13^,^14^,^15^. Gupta *et al*.^12^ investigated the decoding of end-point force applied by monkeys to a joystick. In their study, 96-channel intracortical electrodes were implanted in the M1 area of the two macaque monkeys, the spike trains were obtained and the force parameter was continuously decoded from the firing rate of neurons using a linear decoder. Chen *et al*.^13^ decoded the force profile (not the real force values) from 16-channel ECoG measurements in two monkeys. The monkeys were trained to pull a force sensor and then the normalized force (to maximum values of all the trials) was continuously decoded from ECoG signals. Flint *et al*.^14^ showed the feasibility of decoding isometric force from ECoG signals in human subjects. Ten epileptic subjects were asked to squeeze a force sensor between their fingers and then the applied force was decoded from the ECoG signals. With one exception^15^, to the best of our knowledge LFPs have not been used so far for the continuous decoding of force amplitude. In ref. 15, Milekovic *et al*. showed that both types of griping and load forces can be classified based on the spectral information of 100 LFP channels in two monkeys. They also showed that the force applied to individual fingers during object grasping can be continuously decoded from 100 LFP channels. Here, we decode the continuous force amplitude from the small number of LFP channels (16) in the M1 region of freely moving rats.

## Materials and Methods

### Behavioral task

**Figure 1:**
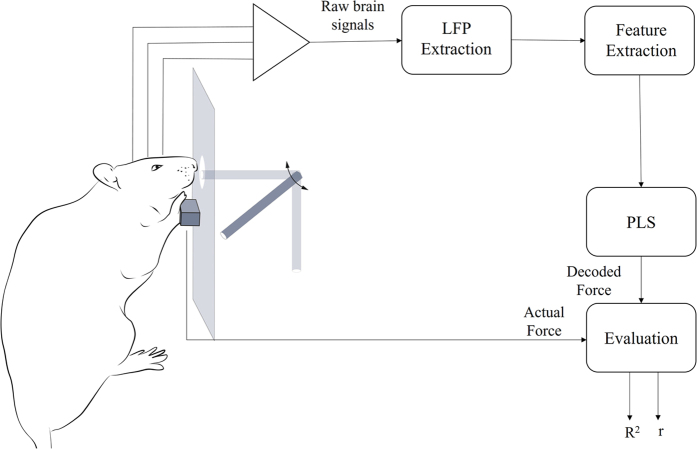
Schematic of force decoding from multichannel LFP data. The rat presses a force sensor and a mechanical arm rotates proportional to the quantity of force and if the applied force by animal’s forelimb reaches to a predefined threshold, the lever will stop on 90 degrees and the animal will have a drop of water as a reward. 16 channels of intracortical signals from the M1 area are recorded simultaneously and the LFP contents are obtained from band-pass filtering (0.1–500 Hz) and down-sampled to 1 kHz. The LFPs are band-pass filtered in 6 frequency sub-bands and a Partial Least Square (PLS) decoder is used to continuously decode force signal from high dimensional feature vector. Coefficient of correlation (r) and coefficient of determination (R^2^) are employed to assess the accuracy of the decoding.

Three male Wistar rats (300–400 g) were used in this study to perform a behavioral task. All rats were trained to control a lever by pressing a key connected to a force sensor (1 DOF load cell) to receive a drop of water as a reward. The force sensor does not rotate during the applying force and was located in the front of the rats 10 cm above the floor of experimental setup. The applied force (0–0.15 N) was linearly mapped to the 0–90 degrees in rotation of a mechanical arm. The animals could only rotate the lever to 90 degrees, but the force values further than 0.15 N are also considered in the analysis. When the applied force reaches a predefined threshold (0.15 N), the lever will stop in 90 degrees angle, and after 1.5 s the animal will receive the water in front of his mouth for 75 ms from the end point of the lever (Fig. 1). The animals were free to press the force sensor at any time and we did not define any cue for the start and end of each trial. Note also that the orientation/position of forelimb during applying force was stable due to the fixation of the force sensor.

### Micro-array implantation

When we ensured that the rats have learned the behavioral task, micro-wire arrays were implanted in the primary motor cortex (M1) area for each rat contralateral to their preferred arm. In this study, all animals used their right hand to perform the task and so the arrays were implanted in the left hemisphere of their brain. All animals were anesthetized by administrating mixture of ketamine (100 mg/kg) and xylazine (10 mg/kg). We monitored the depth of anesthesia by both toe pinching and controlling of respiration rate. Then, animals were mounted on a stereotaxic frame. At the first step of surgery, an incision was made in head skin midline and all of the tissues were removed from the scalp to access the head bone. Then, the Bregma and Lambda points were identified and the desired craniotomy position was marked. Then, one screw was placed in the posterior of Lambda point for connecting the ground wire and 5 extra screws were also mounted on the scalp to stabilize dental acrylic on the head.

The (4 × 4) micro-wire array (Microprobes Inc., Gaithersburg, USA) is constructed from 25 μm Platinum/Iridium Teflon-coated wires (500–800 KΩ). The micro-wire array with inter-wire distance of 500 μm(1.5 × 1.5) was implanted in the forelimb region of the M1. The coordinates of forelimb region were identified using rat brain atlas^16^. The center of the array was implanted in a position 1.6 mm anterior to Bregma, 2.6 mm lateral to the midline and 1.5 mm deep under the surface of the dura mater to cover all the areas of forelimb region^17^,^18^. At the end, the exposed craniotomy and the opened head areas were sealed with dental acrylic. For two days after surgery the animals were intraperitoneally administrated meloxicam (0.2 mg/kg) for pain relief and enrofloxacin (5 mg/kg) every 12 hours for avoiding possible infection. The local ethics committee (The animal care and use committee of Neuroengineering and Neuroscience Research Laboratory, Iran University of Science & Technology) approved all the issues, including training, anesthetization, craniotomy surgery and recovery procedures and all the procedures were in line of NIH protocols for animal research.

### Neural and force data recording

The first session of recording in behaving animals was started 2 weeks after surgery. The animals were placed in the behavioral setup and both brain and force signals were recorded simultaneously while performing the task. The preamplifiers (2*MPA8I) of recording device (USB-ME16, Multichannel system, Germany) were attached to the implanted array connector (Omnetics Connector, USA) and the recorded raw brain signals at sampling rate of 10 KHz were transferred to PC through electrical cable. The spike signals were extracted by band pass filtering of raw brain signals (300–3000 Hz) and then manual thresholding of each channel. Furthermore, to obtain LFP signals the raw brain signals were band-pass filtered (0.1–500 Hz, 4^th^ order Butterworth, band-pass filter, forward and backward) and resampled at the rate of 1 KHz. The force signals were also continuously recorded at the sampling rate of 30 Hz and TTL-synchronization was used to synchronize LFP and force signals. Due to negligible power of force data above 5 Hz, force signals were low-pass filtered at a cutoff of 5 Hz (4^th^ order Butterworth, low-pass filter, forward and backward) and down-sampled to 10 Hz. Because in the designed experimental task the rats were free to push the force sensor at any time, duration of pushing the force sensor by rats has been very small in comparison to the duration that rats did not perform the force-based task. Thus, only data samples corresponding to the task have been considered for the decoding in this study. Although the data were recorded continuously, only the 1.5 s before and 2 s after crossing the applied force from 0.15 N were considered as a trial. Therefore, we obtained one lever press per trial with 3.5 s duration. Then, these extracted trials were concatenated to construct the full training and test sets.

### Continuous decoding of force signal from LFPs

The final goal of this study was to predict the force amplitude applied by the rat forelimb to the force sensor continuously from the multichannel LFPs. In the first step, the common noise in LFP signals was removed using common average referencing (CAR) method^19^. Considering the fact that the recorded brain signals are mixed of cortical signals and a common, noisy term, the noise term can be removed by subtracting the mean of all channels at each time from each one:


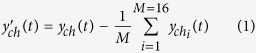


where *y*_*ch*_(*t*) represents the original LFP signal in one channel at time t, 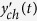 represents the filtered one after applying the CAR filter, and *M* shows the total number of electrodes. In the second step, to extract the meaningful features from the LFP signals, the filtered LFPs using the CAR method were band-pass filtered (4^th^ order Butterworth, band-pass filter, forward and backward) in each channel through the following 6 frequency sub-bands:


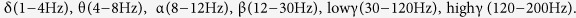


In the next step, to extract the temporal continuous changes related to the force variations in each frequency band, the obtained time series in each frequency band were rectified (absoluted) and then smoothed using a Savitzky-Golay filter (3rd order, 100 ms width). Applying smoothing to the rectified signal provides the envelope of input signal. In this study, unlike conventional methods for smoothing, we used Savitzky-Golay filter as it preserves the local maxima of the original signal better than the others^20^. In the final step of feature extraction, the smoothed features were normalized by subtracting the mean and dividing by the standard deviation values over all the training data.

In the next phase, Partial Least Square (PLS) regression method was used to continuously decode the force signal from the extracted features^21^. The time history of features over 1 s before time t in 0.1 s time-steps were used for force decoding. This leads to a 960 dimensional feature vector (16 channels*6 frequency bands* 10 time lags). To treat with high-dimensional data, PLS regression is a powerful choice due to its ability in both avoiding over-fitting and omitting feature selection procedures. The description of the PLS algorithm has been presented here:

Assume the relationship between the input and output variables based on the linear model *f* = *X β* + *α,* where *X*_*np*_ and *f*_*nl*_ show the input variables (input features) and output response (in our case 1 D force signal), respectively. The parameter n shows the number of samples which is equal to the length of the input signal and p shows the number of available features in input space, respectively. Basically, the PLS method reduces the input features to the smaller sets of uncorrelated components and then applies least square regression on them, instead of the original input data. Furthermore, the PLS method tries to extract components in a way that maximizes the covariance between input and output variables. Assume A (A < p) as the number of optimal components for the prediction of force from the input neural features. PLS iteratively tries to find each component (a = 1, 2, …, A) based on the following steps:

(1) Finding loading weights: These weights show the direction that the covariance between X_a−1_ and f_a−1_ becomes maximum:


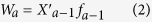


(2) Finding first component: The orthogonal components are estimated using linear coefficients obtained in the previous step:


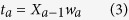


(3) Finding the projection of X (*p*_*a*_) and f (q_*a*_) on the first component *t*_*a*_ by:


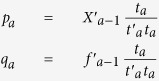


(4) Removing the information of first component from the original input feature X:


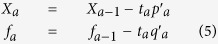


Return to step 1 to find the next component with *X*_*a*_ and *f*_*a*_ as the new input and output variables. These procedures are repeated until finding all components (a = 1, 2, …, A).

(6) In the last step all the computed loading weights, components and loadings are stored in the following matrices:


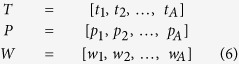


(7) The linear coefficients and bias of PLS decoder are computed based on the following equation:


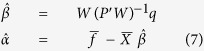


where 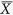 and 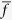 show the average of input and output variables, respectively. Therefore, in our case the regression can be applied between a low dimensional vector (A < 960) and the force signal as output. The final goal of PLS is the estimation of linear coefficients to model the relationship between the extracted feature vector and the output force signal:


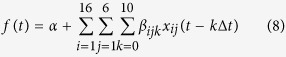


where *f*(*t*) represents the force signal in time *t*, *β*_*ijk*_ is the regression coefficient corresponding to each feature *x*_*ij*_ for *i*^*th*^ channel, *j*^*th*^ frequency band and *k*^*th*^ time lag. *α* is the bias coefficient and temporal lag Δ*t* is equal to 0.1 s here.

To evaluate the accuracy of force decoding from LFPs, 7-fold cross validation method after shuffling the order of trials was used. Thus, the PLS method identified the linear coefficients in each training fold and the decoding performance was evaluated over the test fold which was not used in the training set. Moreover, to select the optimal number of PLS components (A) in each training fold, Wold’s R criterion was used based on the 10-fold cross validation over the training sets^22^:


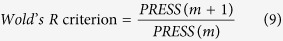


where *PRESS* represents squared error between predicted and observed force signal considering *m* first latent variables for decoding. It is shown that selecting the optimal number of latent variables based on the *Wold’s R* criterion leads to better statistical properties compared with frequent methods that search for minimum *PRESS*^22^. Based on this method, when the defined criterion reaches to a threshold value (0.9), the number of components is considered optimum. We chose the threshold value of 0.9 empirically to achieve the best force decoding performance.

### Performance criteria

We used both coefficient of correlation (*r*) and coefficient of determination (R^2^) to evaluate the accuracy of force prediction from 16-channel LFPs in each test fold. Coefficient of correlation represents the shape similarity between predicted and observed force on the test fold using the following formula:


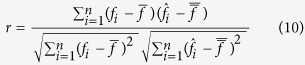


where *f*_*i*_ and 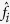 represent i^th^ sample of observed and predicted force, respectively. 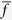
and 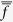
show the mean values of observed and predicted force in the entire of each test fold, respectively. The parameter n shows the total number of samples in each test fold. In addition, the coefficient of determination (*R*^*2*^) is a good criterion to measure the amount of variance in the observed force signal explained by the predicted one which is calculated as following:


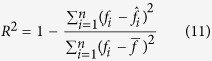


### Contribution of each frequency band and time lag in the force decoding

To investigate the amount of force-related information in each frequency band and time lag, the percentage of contribution of each frequency band and each time lag are calculated based on the following formula:


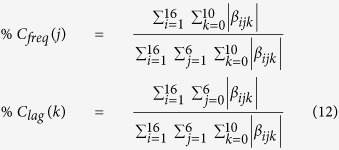


where %*C*_*freq*_(*j*) and %*C*_*lag*_(*k*) are the percentage of contribution of *j*^*th*^ frequency band and *k*^*th*^ time lag in the force decoding, respectively.

## Results

**Figure 2:**
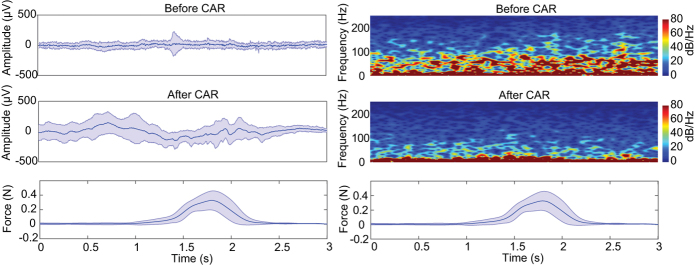
The analysis of the CAR filtering effect on the LFP signals in time and time-frequency domain. Left panel: The mean (bold blue line) ± SD (opaque colored tube) of the LFP signal on channel #11 are drawn over 23 trials in one session recording from rat 3. Right panel: The average spectrogram obtained over the same trials. The mean of (bold blue line) ± SD (opaque colored tube) force signals are shown in the bottom of both panels.

In Table 1, the information of the experiments for each rat is shown. The numbers of trials for each rat (1–3) are 105, 112 and 98, respectively. These trials are obtained from 5, 7 and 5 sessions in each rat respectively spread across 50 days started from two weeks after array implantation. The rats (1–3) have pressed the force sensor 21 (±5.6), 16 (±3) and 19 (±4.2) times on average per session while they were free to press the force sensor at any time. Furthermore, the mean (±SD) of peak force values obtained from all trials in each rat are 0.31 (±0.17), 0.37 (±0.14) and 0.35 (±0.12). Considering the fact that the animal can press the force sensor much further than a predefined threshold (0.15 N), the range of obtained force signals for all trials in different rats (1–3) are 0–0.95, 0–0.79 and 0–0.7 (Fig. 2). It is also important to mention that the negative force values are not considered in the current study, because the animals were not trained to pull the force sensor.

Figure 2 shows an example for the effect of CAR filtering on both temporal and spectro-temporal information of LFP signals. This figure shows the average and standard deviation of LFP signals (session 1, 23 trials, rat3, channel 11) in the time domain before and after applying the CAR filter in a 3 s period before and after one lever press. As can be seen, the CAR filter has increased the variance of some channels, but in some channels this variance has decreased. This shows that the variance parameter is not a proper criterion to evaluate the temporal modulation. But, the results show that applying CAR filter has revealed 27% improvement in decoding performance in terms of R^2^ over all datasets (*ρ* < 0.01, Wilcoxon signed-rank test). These results show that CAR filter has removed the non-force related information from the raw brain signals. The right section of this figure shows the average spectrogram of the same LFP signals (session 1, 23 trials, rat3, channel 11) before and after applying the CAR filter in a 3 s period before and after one lever press. As can be seen, most of the high frequency components of LFPs that may contain artifact sources have been removed.

**Figure 3:**
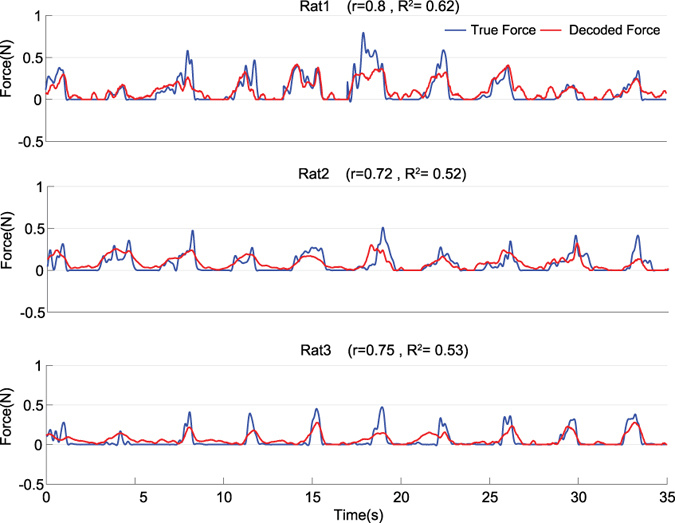
Result of force decoding from 16 channel LFPs in test folds with maximum value of *R*^*2*^ for all the rats. 10 trials of these test folds have been shown as an example.

**Figure 4:**
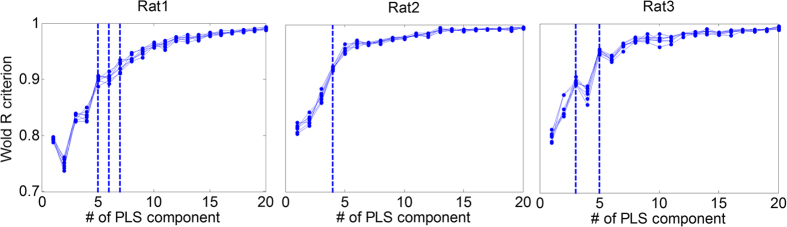
Selecting the optimal number of PLS components based on the Wold’s R criterion for all 7 folds in each rat dataset.

**Figure 5:**
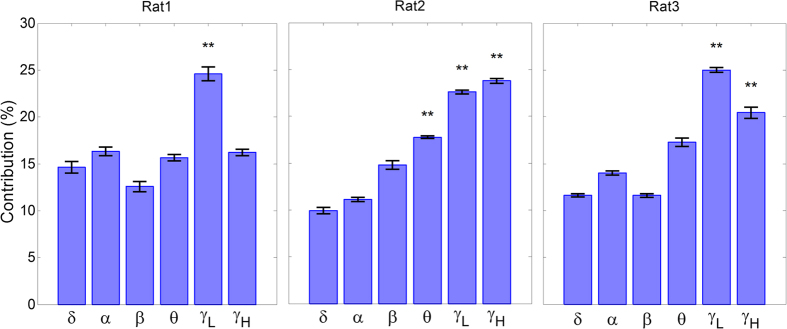
Contribution of each frequency band in force decoding. Each bar shows the mean and standard error of contribution weights obtained from 7 folds. The frequency bands with contribution significantly greater than the mean value of all contributions are marked with asterisks (*ρ* < 0.01, Wilcoxon signed-rank test).

**Figure 6:**
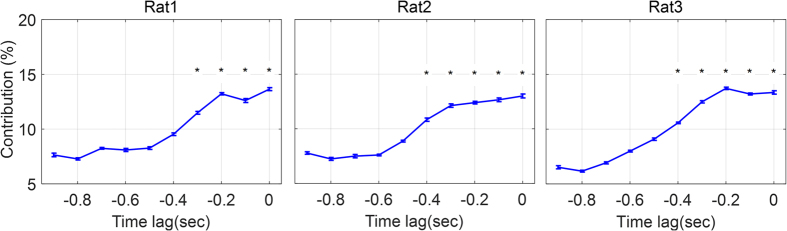
The contribution of each time lags in force decoding. Each bar shows the mean and standard error of contribution weights obtained from 7 folds. The time lags with contribution significantly greater than the mean value of all contributions are marked with asterisks (*ρ* < 0.01, Wilcoxon signed-rank test).

The results of force decoding from 16 channel LFPs for all 3 rats in each of 7 test folds are represented in Table 2. As it can be seen, the mean (±SD) of *r* and *R*^*2*^ values obtained from all 7-test folds for each rat (1–3) are (*r* = 0.68 ± 0.06, *R*^*2*^ = 0.45 ± 0.1), (*r* = 0.64 ± 0.04, *R*^*2*^ = 0.41 ± 0.08) and (*r* = 0.67 ± 0.05, *R*^*2*^ = 0.42 ± 0.08), respectively. Figure 3 shows 35 s (10 trial) of force decoding in a test fold with the highest value of *R*^*2*^. The *R*^*2*^ value in these best folds for each rat (1–3) is 0.63, 0.52 and 0.53, respectively. The mean (±SD) optimal number of components based on the *Wold’s R* criterion for all 7 folds is 5.7 (±0.81) for rat 1, 4 (±0) for rat 2 and 4.71 (±0.75) for rat 3 (Fig. 4). The obtained values show that the number of selected PLS components for force decoding is much smaller than the overall feature dimension (960). Figure 5 shows the contribution of each frequency band corresponding to 7 folds of each rat dataset. As it can be seen, in all 3 datasets, the contribution of low gamma band (30–120 Hz) has been significantly greater than the mean contribution of all frequency bands (Wilcoxon signed-rank test). Figure 6 shows the temporal contribution of different time lags obtained from 7 folds in each dataset. As can be seen the contribution of temporal offsets (Δ*t*) between 0–300 ms have been significantly greater than the mean contribution of all time lags (Wilcoxon signed-rank test). It should be noted that backward filtering may also introduce some temporal leakage in the information, so the temporal contribution may have changed after applying backward filtering.

**Figure 7:**
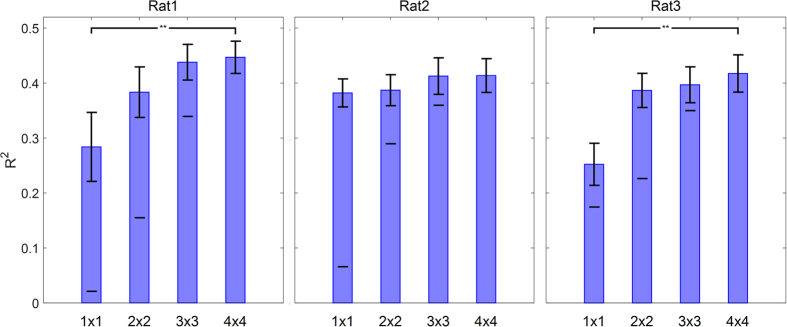
The analysis of force decoding performance corresponding to different channel groups. Each bar shows the mean and standard error of R^2^ values obtained from 7 folds of best channel combination. The mean of R^2^ values corresponding to the worst result in each combination is shown with a black line in each bar. The channel groups with significant difference in mean R^2^ are shown with asterisks (*ρ* < 0.05, Wilcoxon rank sum test).

To investigate the decoding performance of different channel groups, 4 combinations of channels including 1 × 1, 2 × 2, 3 × 3 and 4 × 4 were selected for the analysis. In 1 × 1 cases, the decoding performance of each channel is computed separately. In 2 × 2 cases, the decoding performance of 4 adjacent channels is computed and so 9 different 2 × 2 configurations have been considered separately. In 3 × 3 cases, 9 adjacent channels are selected and so 4 different 3 × 3 configurations have been considered separately. In 4 × 4 case, all channels have been used for the force decoding. In Fig. 7, the best result of force decoding in each of 4 conditions resulted from 7-test folds for all rats is shown. As can be seen, in all 3 rats the decoding performance of 2 × 2, 3 × 3 and 4 × 4 were not significantly different for the best results (*ρ* > 0.3, Wilcoxon rank sum test).

**Figure 8:**
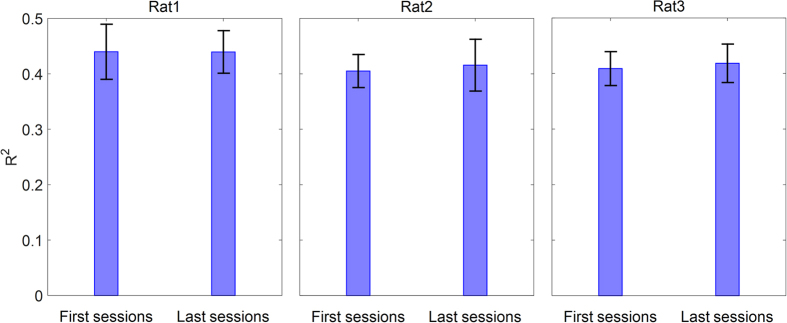
The analysis of stability of force decoding performance in different sessions. Left bars show the mean and standard error of R^2^ values corresponding to trials obtained from experiment sessions on days 15 to 30 after array implantation. Right bars show the mean and standard error of R^2^ values corresponding to trials obtained from experiment sessions on days further than 45 after array implantation.

We also investigated the variability of force decoding performance across different sessions. Because the number of trials in some sessions was low and different across days, 40 trials corresponding to the period 30 days after array implantation are selected as first sessions and 40 trials corresponding to the period 45 days after array implantation are selected as last sessions. In this way, the interval between trials in each group would be at least 15 days. The mean (±SD) of *R*^*2*^ values obtained from all 7-test folds in the first sessions are 0.43 (±0.13) for rat 1, 0.40 (±0.08) for rat 2, 0.40 (±0.08) for rat 3 and in the last sessions are 0.43 (±0.10) for rat 1, 0.41 (±0.12) for rat 2 and 0.41 (±0.09) for rat 3. Figure 8 demonstrates that the decoding of force amplitudes has not significantly changed in different sessions (*ρ* > 0.7, Wilcoxon rank sum test). Moreover, to show the force decoding stability, we trained the decoder using 40 trials of first sessions related to 15–30 days after array implantation and then tested decoding performance using 40 trials of the last sessions related to >45 days. The R^2^ values in each rat dataset (rat 1–3) obtained from the evaluation on the last sessions were 0.30, 0.33 and 0.31, respectively.

## Discussion

In the current study, we showed that the force amplitude can be continuously decoded from a small number of channel LFPs. As far as we know, this is the first study on the continuous decoding of the forelimb force form LFPs in freely moving rats. In ref. 23 the force parameter was continuously extracted from the neural signals recorded from the corticospinal tract (CST) of the rat spinal cord. However, designing a spinal cord computer interface does not seem a practical human-based system since firstly the mechanical stability of implanted arrays in the spinal cord is a crucial issue and secondly, this technology cannot be used for tetraplegia or ALS patients due to lack of receiving control signals from cortex^23^. Furthermore, in experimental setup of the aforesaid study, the force sensor was located on the floor of the experimental setup. Thus, the measured force may not be purely resulted from the applied force by animal forelimb and the weight of the animal may be contributed in the measured force. In our study, at the stage of designing the experimental task we considered two important issues. Firstly, we tried to diminish the forelimb movements by using a load cell sensor as a rigid body that its rotation around its axis is negligible. Although, the orientation/position of forelimb on the force sensor may change across trials, in each trial the orientation/position of forelimb has remained stable due to fixation of the force sensor. Therefore, the applied force can be considered as an isometric force. Secondly, we located the force sensor in a height that is not too high (to raise and push it), nor low (to use its body weight) in front of the rats (Fig. 1). In this design, the rats have to apply only a perpendicular force to the sensor to move the mechanical lever and receive the reward. In the current study animal can move freely, so all the body parts of the animal, including neck and hind limb parts and also power line move during the task. Fortunately, with intracortical recording the electrodes can be locally implanted in the desired location for decoding (forelimb area in this study). Therefore, it is unlikely that neural activity of other cortical areas like neck region affects decoding procedure. In addition, unlike EEG based studies that have proved the effect of somatosensory feedbacks on decoding^24^, using the intracortical techniques with higher spatial resolution can diminish the effect of sensory inputs (muscle spindles or Golgi tendons) in the force decoding. However, this impact cannot be completely ignored due to the projection of proprioceptive information to the motor cortex^25^. This issue should not be considered as a negative factor in the force decoding, because these sensory inputs might also become available in real BMI systems^26^. Furthermore, in comparison to ECoG based recordings^27^, intracortical recordings benefits from a higher spatial resolution which can be considered as a powerful advantage in decoding capability^4^.

In this study, we used PLS decoder for force decoding because of its popularity working with multi-dimensional data. In this method the most informative components related to the output force amplitude are extracted from high dimensional neural features. We also used *Wold’s R* criterion to choose the optimal number of task-related components. Based on this criterion, the components that significantly improve the decoding performance are selected in the PLS model. So, the optimal number of components obtained from all 7 test folds for all 3 rats has been significantly less than the overall dimension of input features (4.8 ± 0.13 components on average for all 3 rat datasets out of 960 available features).

The comparison of force decoding accuracy from LFP signals in this study with other BMI studies shows that the obtained performances in terms of *r* and *R*^*2*^ are fairly comparable to the previous works [15, 14, 22]. In ref. 15 the applied force amplitudes to the individual fingers during object grasping were continuously decoded from 100-channel LFPs with mean accuracy of 0.21 over all dataset. But, we obtained better accuracy (*R*^*2*^ = 0.42 in average over all dataset) with only 16 channels of LFP. This better accuracy can be resulted either from good performance of the PLS decoding method or from accurately locating the implanted array in forelimb area. However, both somatotopic organization (overlap of finger representation in the motor cortex) and co-activation of finger muscles make decoding of force from individual fingers more complex than the decoding of force applied to the object similar to our study. Furthermore, Flint *et al*.^14^ could decode isometric grasping force with an average accuracy of *R*^*2*^ = 0.6 from 10 human ECoG signals. Furthermore, in ref. 23 isometric force parameter were continuously decoded from 32 electrodes implanted in the cortical spinal tract of the rat spinal cord with accuracy of *r* = 0.66 and *R*^*2*^ = 0.44 which is comparable to our results.

The investigation of each frequency band contribution in force decoding in our study is also in agreement with other studies. In this study, the results show that the contribution of low gamma band frequency (30–120 Hz) in force decoding for all 3 rat datasets have been significantly greater than other frequency bands. In ref. 14 it is shown that (70–115 Hz) band of human ECoGs has been the most informative frequency band for continuous force decoding. Also, in ref. 4 it is shown that (70–250 Hz) band frequency of LFPs contains great information about movement. A study by Milekovic *et al*.^15^ also shows that the high frequency band (80–250) is highly modulated with the force signal. In refs 9 and 28 it is also shown that information about movement direction is significantly located in the gamma band (>60 Hz). However, several studies have shown that the task-based information on gamma bands of LFPs is related to the spiking activity^29^,^30^,^31^, our results complement with them representing that high-gamma bands have had a lower contribution in the force decoding in comparison to low-gamma content of LFPs. Therefore, it is unlikely that the obtained results in this study have been merely resulted from the spiking activities. This inference is in agreement with a study by^4^ showing that the decoding of movement parameters from LFPs has remained stable regardless absence of spiking activities in the same electrode selected for decoding.

We investigated how the decoding performance may change with the smaller number of channels. The results showed that by selecting 2 × 2 or 3 × 3 configuration the force parameter can be decoded with performance almost equal to considering all the channels. However, it is also important to notice that the decoding performance is more dependent on the density of channels in the desired region than the number of electrodes. For example, in rat 2 (Fig. 7), with only one electrode it is possible to decode the force parameter with performance not significantly different from considering all the channels. Achieving high performance with a small number of channels can be considered as an important achievement, specifically in the wireless BMI systems due to lower power consumption.

We also investigated the stability of force decoding performance through the time. The results showed that, although the interval between the trials in the first sessions related to 15–30 days after array implantation and the last sessions related to >45 days (Fig. 8) has been at least 15 days, the decoding performance has not significantly changed. Thus, we can conclude that the decoding performance will remain stable through the time by calibration of decoder for each session. Furthermore, we performed an analysis to investigate the stability of kinetic information in LFPs by training PLS decoder with the trials related to 15–30 days after array implantation and testing with the trials related to >45 days. Although, the obtained results based on this analysis were acceptable in comparison to other studies, calibrating the decoder at the beginning of each session would increase the decoding performance.

As a future work, we plan to control an external device like the mechanical lever using the decoded force from LFPs. On that stage, the animal presses the force sensor, but the lever will be rotated based on the decoded force from LFPs. It would be interesting for us to investigate if the decoding performance can be changed in real time brain control. Based on the results obtained from other studies^32^,^33^, we expect that the force decoding performance amplify when the rats learn to control the lever using their brain in online mode.

## Conclusion

In conclusion, this study is the first step towards an online decoding of force from LFP signals. Our study presents two important novelties with respect to other studies in BMI systems. Firstly, we used only 16 channels of LFPs for continuous decoding of force in comparison to other invasive BMI studies that use high density of electrodes (>64) for decoding of kinematic or kinetics parameters. Secondly, we tried to design a task similar to real-life BMIs as animals can move freely during performing the task.

## Additional Information

**How to cite this article**: Khorasani, A. *et al*. Continuous Force Decoding from Local Field Potentials of the Primary Motor Cortex in Freely Moving Rats. *Sci. Rep.* **6**, 35238; doi: 10.1038/srep35238 (2016).

## Figures and Tables

**Table 1:** Details of the trials in each rat experiment.

	*Rat1*	*Rat2*	*Rat3*
*Number of trials*	*105*	*112*	*98*
*mean of peak force* (*N*)	*0.31* ± *0.17*	*0.37* ± *0.14*	*0.35* ± *0.12*
*Force values range* (*N*)	*0–0.95*	*0–0.79*	*0–0.71*

**Table 2:** Results of force decoding in all 7 test folds for all rats.

*Test Fold*	*Rat1*		*Rat2*		*Rat3*	
	*r*	*R*^*2*^	*r*	*R*^*2*^	*r*	*R*^*2*^
*1*	*0.74*	*0.49*	*0.58*	*0.33*	*0.70*	*0.46*
*2*	*0.64*	*0.33*	*0.65*	*0.42*	*0.63*	*0.40*
*3*	*0.70*	*0.49*	*0.67*	*0.44*	*0.59*	*0.29*
*4*	*0.67*	*0.44*	***0.72***	***0.52***	***0.75***	***0.53***
*5*	***0.80***	***0.62***	*0.62*	*0.40*	*0.71*	*0.49*
*6*	*0.57*	*0.33*	*0.65*	*0.42*	*0.62*	*0.38*
*7*	*0.66*	*0.43*	*0.61*	*0.37*	*0.68*	*0.44*
*Mean* (±*SD*)	*0.68* ± *0.06*	*0.45* ± *0.1*	*0.64* ± *0.04*	*0.41* ± *0.08*	*0.67* ± *0.05*	*0.42* ± *0.08*
